# Connexin and Pannexin-Based Channels in Oligodendrocytes: Implications in Brain Health and Disease

**DOI:** 10.3389/fncel.2019.00003

**Published:** 2019-01-29

**Authors:** Sebastián Vejar, Juan E. Oyarzún, Mauricio A. Retamal, Fernando C. Ortiz, Juan A. Orellana

**Affiliations:** ^1^Mechanisms of Myelin Formation and Repair Laboratory, Instituto de Ciencias Biomédicas, Facultad de Ciencias de la Salud, Universidad Autónoma de Chile, Santiago, Chile; ^2^Departamento de Neurología, Escuela de Medicina and Centro Interdisciplinario de Neurociencias, Facultad de Medicina, Pontificia Universidad Católica de Chile, Santiago, Chile; ^3^Centro de Investigación y Estudio del Consumo de Alcohol en Adolescentes, Pontificia Universidad Católica de Chile, Santiago, Chile; ^4^Centro de Fisiología Celular e Integrativa, Facultad de Medicina, Clínica Alemana Universidad del Desarrollo, Santiago, Chile; ^5^Department of Cell Physiology and Molecular Biophysics, and Center for Membrane Protein Research, Texas Tech University Health Sciences Center, Lubbock, TX, United States

**Keywords:** connexons, oligodendrocytes, pannexons, hemichannels, gap junctions, demyelinating neuropathy

## Abstract

Oligodendrocytes are the myelin forming cells in the central nervous system (CNS). In addition to this main physiological function, these cells play key roles by providing energy substrates to neurons as well as information required to sustain proper synaptic transmission and plasticity at the CNS. The latter requires a fine coordinated intercellular communication with neurons and other glial cell types, including astrocytes. In mammals, tissue synchronization is mainly mediated by connexins and pannexins, two protein families that underpin the communication among neighboring cells through the formation of different plasma membrane channels. At one end, gap junction channels (GJCs; which are exclusively formed by connexins in vertebrates) connect the cytoplasm of contacting cells allowing electrical and metabolic coupling. At the other end, hemichannels and pannexons (which are formed by connexins and pannexins, respectively) communicate the intra- and extracellular compartments, serving as diffusion pathways of ions and small molecules. Here, we briefly review the current knowledge about the expression and function of hemichannels, pannexons and GJCs in oligodendrocytes, as well as the evidence regarding the possible role of these channels in metabolic and synaptic functions at the CNS. In particular, we focus on oligodendrocyte-astrocyte coupling during axon metabolic support and its implications in brain health and disease.

## Introduction

Oligodendrocytes are the myelin-forming cells of the central nervous system (CNS). They were first described and characterized as small cells with many branches distributed ubiquitously within the CNS in a close spatial relationship with axons (del Río-Hortega, 1922; Ramón y Cajal, 1925). Oligodendrocyte processes wrap the axon at different points, forming the multilamellar structure of myelin, which is organized in sheaths along the axonal axis. Myelin sheaths enable axons for saltatory conduction accounting for the speed-up of action potential conduction in neurons (Tasaki, 1939; Nave and Werner, 2014). By exerting this main function, oligodendrocytes largely contribute to establish the conduction properties of neural circuits, leading to the proper integration of electrical signaling at the CNS. Consequently, in diseases characterized by the loss of myelin—such as multiple sclerosis (MS)—patients show several neurological impairments, revealing the decisive contribution of oligodendrocytes to brain function (Franklin and Ffrench-Constant, 2008, 2017).

In addition to their key role on saltatory conduction, oligodendrocytes participate in other important functions ranging from metabolic supply and ion buffering to setting survival and excitability properties of neurons (Menichella et al., 2006; Hamada and Kole, 2015; Battefeld et al., 2016; Saab et al., 2016; Philips and Rothstein, 2017). The plastic nature of oligodendrocytes relies not only in their ability to fulfill diverse functions, but also on the fact that they conform heterogeneous populations with different properties. Indeed, according to their morphological complexity, size and distribution, oligodendrocytes can be grouped into four subtypes (del Río-Hortega, 1922; Pérez-Cerdá et al., 2015). Type I and II represent the less abundant population, which myelinates mainly small axons in the *corpus callosum* and the cortex, while type III and IV are associated to the myelination of larger axons in the spinal cord (Dimou and Simons, 2017). A different grouping criterion—based mainly in the oligodendrocyte location around neurons—identify another subtype of oligodendrocytes, named *satellite* oligodendrocytes (Ludwin, 1979; Takasaki et al., 2010; Battefeld et al., 2016). Satellite oligodendrocytes are normally located at perineural gray matter regions, and although they have not been extensively characterized, evidence indicates that this population could play also trophic roles by giving metabolic support and extracellular K^+^ buffering to neurons (Szuchet et al., 2011; Battefeld et al., 2016). Most of the current literature characterize satellite oligodendrocyte as a non-myelinating population, however, recent studies have shown that under certain conditions (i.e., demyelination) they actually synthetize myelin, forcing to revisit the current notion of *satellite* oligodendrocytes usually based on their inability to synthetize myelin (Szuchet et al., 2011; Battefeld et al., 2016).

In the nervous system, oligodendrocytes interact with several neighboring cells by multiple mechanisms. For instance, they sense growth factors and cytokines released by surrounding astrocytes and microglial cells (Ishibashi et al., 2006; Miron et al., 2013), particularly, under pathological conditions, where these signals can induce the maturation of oligodendrocyte precursor cells (OPCs) into oligodendrocytes or stabilize their mature phenotype (Fulmer et al., 2014; Hammond et al., 2015; Miyamoto et al., 2015). Similarly, oligodendrocytes and OPCs can respond to neural activity by detecting neurotransmitters through the activation of ionotropic and metabotropic receptors (i.e., AMPARs, NMDAR, mGluRs, GABAxRs and nAChRs; Bergles et al., 2000; Lin and Bergles, 2004; Kukley et al., 2007; Vélez-Fort et al., 2009; Li et al., 2013; Orduz et al., 2015; Wake et al., 2015; Saab et al., 2016; Fields et al., 2017). Commonly, glial cells communicate each other and with neurons *via* the release of bioactive molecules called gliotransmitters (Araque et al., 2014). Although it is unclear whether oligodendrocytes are endowed with the machinery to allow the release of gliotransmitters, they form extensive functional interactions among them and with astrocytes through special structures called gap junctions (GJs).

GJs result from the accumulation of intercellular channels in areas of close apposition between two plasma membranes of adjacent cells. These channel-called GJs channels (GJCs)-favor the direct intercellular exchange of metabolites (e.g., ADP, glucose, glutamate and glutathione), second messengers (e.g., cAMP and IP_3_) and ions, allowing the cell-cell spread of electrotonic potentials in excitable and non-excitable tissues (Leybaert et al., 2017). When two hemichannels from adjacent cells align with each other in appositional membranes, they make a full intercellular GJC (Saez et al., 2003). Each hemichannel is constituted by the oligomerization of six monomers of connexins, a highly conserved protein family encoded by 21 genes in humans and 20 in mice, with orthologs in other vertebrate species (Abascal and Zardoya, 2013). Recent data indicates that along with constitute the building blocks of GJCs, hemichannels in nonjunctional membranes may serve as diffusional routes for ion and small molecules between the cytoplasm and the extracellular compartment (Montero and Orellana, 2015). Fifteen years ago, a new gene family encoding a set of three membrane proteins termed pannexins was discovered (Bruzzone et al., 2003). Despite that pannexins do not share significant amino acid sequences with connexins, both families have analogous secondary and tertiary structures (Abascal and Zardoya, 2013). Most of findings implicate that pannexins are capable to form single membrane channels, termed pannexons, that have some similarities with hemichannels (Sosinsky et al., 2011). Under healthy conditions, hemichannels and pannexons underpin the release of gliotransmitters (e.g., ATP, glutamate, D-serine, lactate), acting as crucial players in multiple brain processes such as synaptic transmission, neuronal oscillations, glucose sensing, ischemic tolerance and fear memory consolidation (Lin et al., 2008; Orellana et al., 2012; Stehberg et al., 2012; Chever et al., 2014; Roux et al., 2015; Meunier et al., 2017). Nonetheless, the permanent and exacerbated activity of these channels might operate as a cornerstone in the prelude and development of homeostatic imbalances detected in diverse neuropathological diseases (Orellana et al., 2016).

In this work, we review and discuss the evidence sustaining the possible role of oligodendrocyte GJ coupling in the coordination of metabolic support for neuronal activity, as well as its participation in demyelination processes observed in different diseases. In addition, we overview the recent evidence arguing for the functional expression of hemichannels and pannexons in oligodendrocytes and OPCs and how this may impact not only their normal metabolism, but also their survival during inflammatory scenarios.

## Oligodendrocyte-Oligodendrocyte Gap Junctional Communication and Their Contribution to Health and Disease

### General Characteristics of Connexin Expression in Oligodendrocytes

**Figure 1:**
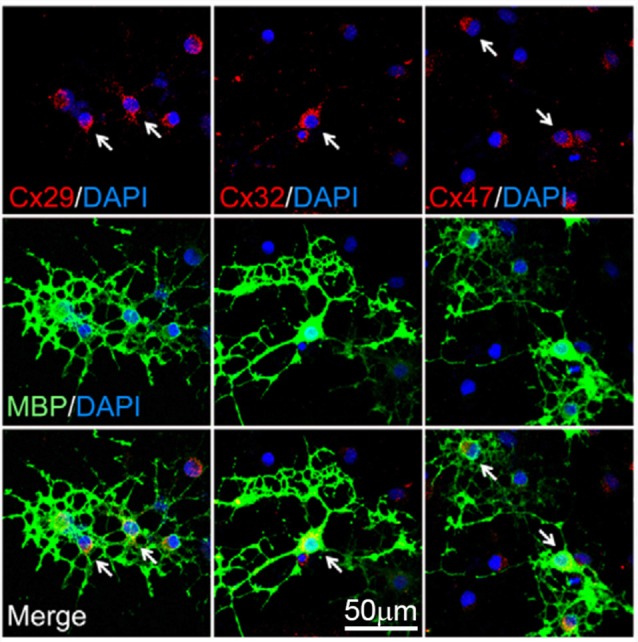
Expression of connexins by oligodendrocytes. Representative fluorescence images depicting the double immunostaining of connexin 29 (Cx29; red, left panels), Cx32 (red, middle panels) and Cx47 (red, right panels) with the oligodendrocyte-specific bio-marker myelin basic protein (MBP, green) and DAPI staining (blue) by mature cultured oligodendrocytes after 6 days *in vitro*. Arrows highlight representative MBP-positive cells. Adapted, with permission, from Niu et al. (2016).

Oligodendrocytes have been reported to express connexin 29 (Cx29), Cx32, Cx45 and Cx47 (Figure 1; Dermietzel et al., 1989; Micevych and Abelson, 1991; Nagy et al., 2003; Li et al., 2004). Although the latter could imply that any of these connexins may form GJCs in oligodendrocytes, early studies showed contradictory experimental data, revealing the complexity of detecting GJ structures in these cells (Orthmann-Murphy et al., 2008; Nualart-Marti et al., 2013). Pioneering studies using freeze-fracture electron microscopy (FEM) in rat embryonic cultures described that contrary to that found in astrocytes, oligodendrocytes do not form GJCs between them (Massa and Mugnaini, 1982). Comparable results were obtained from FEM experiments in adult cat brain and spinal cord, were astrocyte-astrocyte and astrocyte-oligodendrocyte GJs were observed, but inter-oligodendrocyte GJs were not detected (Massa and Mugnaini, 1982). Similarly, freeze-fracture of freshly isolated oligodendrocytes from adult lamb brains, showed that GJs between oligodendrocytes were small and infrequent (Massa et al., 1984).

**Figure 2:**
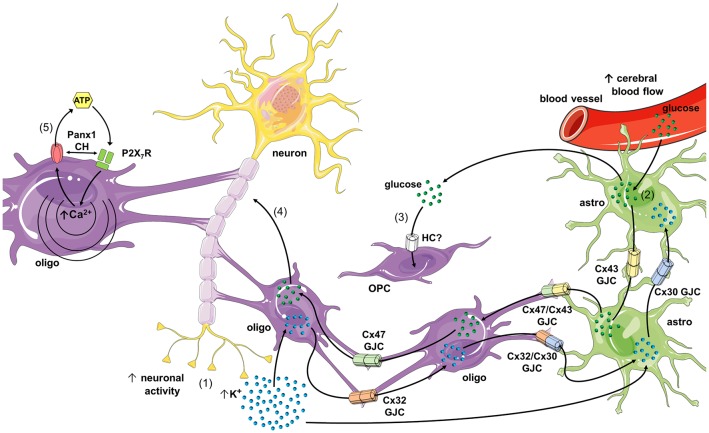
Schematics of possible roles of connexin- and pannexin-based channels in oligodendrocyte function and dysfunction. During high rates of neuronal activity K^+^ accumulates in the extracellular space, and then is taken up by oligodendrocytes and astrocytes through the inwardly rectifying K^+^ channel (Kir) 4.1 and/or Na^+^/K^+^-pumps (1). K^+^ that concentrates inside oligodendrocytes and astrocytes diffuses to the panglial syncytium *via* homocellular and heterocellular gap junction channels (GJCs), a process termed “spatial K^+^ buffering.” In parallel, neuronal and astroglial signaling (e.g., ATP and glutamate) could activate endothelial P2 and NMDA receptors (NMDARs), respectively, leading to increased free [Ca^2+^]_i_ and further vasodilation of blood vessels (not depicted). The latter increases cerebral blood flow and further uptake of glucose by astrocytic endfeet (2). Glucose diffuses through astrocytes and oligodendrocytes *via* homocellular and heterocellular GJCs and then can be metabolized to lactate by astrocytes, and both can be released into the extracellular space. In addition, glucose is taken up by oligodendrocyte precursor cells (OPCs; *via* an unknown hemichannel) and neurons, which possibly modulate oligodendrocyte differentiation and maturation (3), as well as axonal function (4), respectively. In pathological scenarios, the opening of Panx1 channels may lead to the release of ATP from oligodendrocytes (5), resulting in the activation of P2X_7_ receptors (P2X_7_Rs) and further triggering of a self-perpetuating mechanism of cell damage, in which high levels of [Ca^2+^]_i_ and direct protein-protein interaction could reactivate pannexons. Part of this schematics was done with support of the free online Servier Medical Art repository (https://smart.servier.com/).

Contrasting the above findings, Kettenmann et al. (1983) described the successful coupling between oligodendrocytes from 2-week old mouse explants, as measured by dye transfer and electrophysiological recordings. An interesting result of this work was that about 34% of oligodendrocytes were electrically coupled, whereas the 70% exhibited positive Lucifer yellow diffusion to another oligodendrocyte. Indeed, dye-coupled oligodendrocytes were seen as far as 300 μm from the original injected cell (Kettenmann et al., 1983). This study was further supported by findings indicating that long term cultured oligodendrocytes from adult bovines establish GJ-like structures between them, as observed through electron microscopy (Norton et al., 1983). The apparent contradiction in which early studies described virtually no GJ-like structures in oligodendrocytes vs. other findings showing the opposite, probably relies on technical discrepancies, as well as the complex diversity of oligodendrocytes and their differential pattern of connexin expression. For instance, oligodendrocytes expressing O1 or O4 do not display dye or electrical coupling, while those expressing O10 show almost 40% coupling (Von Blankenfeld et al., 1993). Additionally, in slices from rat spinal cords, about 18% of oligodendrocytes in the gray matter are coupled, as showed by Lucifer yellow and neurobiotin coupling assays, whereas oligodendrocyte from white matter are not (Pastor et al., 1998). The latter findings suggest that oligodendrocyte gap junctional communication may change depending on the CNS area. Nowadays, the accumulating *in vivo* and *in vitro* evidence indicates that oligodendrocytes are coupled to each other through homotypic GJCs formed mainly by Cx47 and secondarily by Cx32 (Figures 1, 2; Maglione et al., 2010; Wasseff and Scherer, 2011).

Myelin sheaths of both Schwann cells and oligodendrocytes are *intraconnected* through GJs formed by Cx32, allowing the flux of ions and molecules from the perinuclear cytoplasm to the rest of the cell (Balice-Gordon et al., 1998; Nagy et al., 2003; Kamasawa et al., 2005). In fact, cultured oligodendrocytes form tight junctions and GJCs between their myelin sheaths, reassembling what occur *in vivo* (Gonatas et al., 1982). Although myelin sheaths express Cx29 (Cx31.3 in humans; Sargiannidou et al., 2008) and Cx32 (Nagy et al., 2003; Li et al., 2004; Nualart-Marti et al., 2013), they do not colocalize with each other. Particularly, Cx29 is detected in the inner/adaxonal membrane (opposing the axonal membrane) of small myelinated fibers (Altevogt et al., 2002), whereas Cx32 is localized from the soma towards the abaxonal membrane of primarily large myelinated sheaths and among contiguous layers of myelin membrane at the paranode (Kleopa et al., 2004; Kamasawa et al., 2005). Of note, despite its localization in the cell body of oligodendrocytes, Cx29 seems to form hemichannels, likely in the adaxonal membrane, rather than function as GJC (Altevogt et al., 2002; Altevogt and Paul, 2004; Kamasawa et al., 2005; Ahn et al., 2008; Orthmann-Murphy et al., 2008). In agreement with this idea, Cx31.3-the human ortholog of murine Cx29-exhibit functional features compatible with the formation of hemichannels, but not GJCs (Sargiannidou et al., 2008). In the white and gray matter, Cx47 localizes in the cell body and initial processes of oligodendrocytes, as well as in the abaxonal membrane of myelinated fibers (Nagy et al., 2003; Altevogt and Paul, 2004; Kleopa et al., 2004; Kamasawa et al., 2005). In those cell areas Cx47 may form homotypic Cx47/Cx47 GJCs among oligodendrocytes and heterotypic GJCs with astrocytes expressing Cx43 (see next section; Figure 2; Wasseff and Scherer, 2011; Fasciani et al., 2018).

### Functional and Dysfunctional Coupling Among Oligodendrocytes

Astrocytes are extensively coupled to each other through homotypic GJCs composed by Cx30 or Cx43 (Giaume et al., 1991; Nagy et al., 2001). For a long time, they were considered as the major protagonists in the buffering of extracellular K^+^ from the synaptic cleft (Walz, 2000). Nonetheless, recent evidence indicates that oligodendrocytes along with astrocytes may form a “panglial syncytium” connected through GJs, constituting the principal pathway for the long-distance siphoning of K^+^ from the juxtaparanodal periaxonal space to vasculature or the cerebrospinal fluid (Menichella et al., 2006; Rash, 2010). Oligodendrocytes form heterocellular GJs with astrocytes through heterotypic channels of Cx47 with Cx43, but also by Cx32/Cx30 GJCs depending on the anatomical area (Figure 2; Orthmann-Murphy et al., 2007b; Maglione et al., 2010; Magnotti et al., 2011; Wasseff and Scherer, 2011; Tress et al., 2012; Fasciani et al., 2018). This unequal connexin combination of GJCs could derive in a unidirectional rather than a bidirectional ion and molecular interchange between oligodendrocyte and astrocytes (Flagg-Newton and Loewenstein, 1980). This may imply the existence of a directional diffusion barrier that might account for the spatial buffering of periaxonal K^+^ as well as for metabolic coupling and signaling between these cell types. Consistent with this notion, Cx32/Cx30 GJCs show an evident fast ionic rectification depending on the polarity of transjunctional voltage (Orthmann-Murphy et al., 2007b), whereas Cx47/Cx43 GJCs display ionic and chemical rectification, which result in a directional permeability barrier for the movement of ions and molecules from cells expressing Cx47 to those endowed with Cx43 (Fasciani et al., 2018). Interestingly, electron microscopy and freeze-fracture replica immunogold labeling revealed that voltage-gated K^+^ channels Kv1.1/Kv1.2 form densely packed “rosettes” that are exactly aligned with Cx29 hemichannels in the surrounding juxtaparanode myelin collars and along the inner mesaxon (Rash et al., 2016). Whether Cx29 functionally docks with Kv1.1/Kv1.2 to constitute a full myelin-axon channel involved in K^+^ buffering remains to be elucidated. If the existence of this new entity is confirmed, it will open a new line of the study in the field, as the discover of pannexins did years ago.

An additional key function of oligodendrocytes is their ability to provide metabolic support to the axonal compartment of neurons (Saab et al., 2016; Philips and Rothstein, 2017; Meyer et al., 2018). Although the underlying mechanisms of this phenomenon are not completely understood, GJCs between oligodendrocytes and astrocytes, as well as direct oligodendrocyte-neuron gap junctional communication have been pointed out as major mediators on this process (Nualart-Marti et al., 2013; Philips and Rothstein, 2017; Meyer et al., 2018). A recent study, from Niu et al. (2016) attempted to evaluate whether connexin-based channels contribute to glucose trafficking pathways in oligodendroglial cells. They observed that a glucose analog can diffuse through oligodendrocyte-astrocyte but not OPC-astrocyte GJs, suggesting the presence of a panglial metabolic route that may depend on the developmental stage of the oligodendroglial lineage (Niu et al., 2016). In agreement with these data, cells expressing the surface ganglioside A2B5 and OPCs are not dye or electrically coupled to astrocytes whereas mature oligodendrocytes do couple with astrocytes (Bergles et al., 2000; Lin and Bergles, 2004; Xu et al., 2014). Moreover, oligodendrocytes have the ability to supply lactate or glucose to maintain axonal function, depending on the white matter region (Saab et al., 2016; Meyer et al., 2018). Interestingly, experiments carried out on the *corpus callosum* found that in knockout animals for Cx47, oligodendrocytes lose the ability to maintain axonal function (Meyer et al., 2018). Moreover, this effect was astrocyte independent, suggesting that proper axonal function requires a direct energy supply from oligodendrocytes by a mechanism that may involve the opening of Cx47-based channels (Figure 2; Meyer et al., 2018). Accordingly, major modifications on GJ-based connectivity have been reported for both oligodendrocytes and astrocytes under demyelinated conditions, where an impaired metabolism is expected (Kleopa et al., 2010; Markoullis et al., 2012, 2014).

How does the disruption of GJ communication among oligodendrocytes contribute to CNS diseases? Charcot-Marie-Tooth (CMT) disease is importantly correlated with mutations in Cx32 gene (Fairweather et al., 1994; Kleopa et al., 2010; Table 1). This neuropathology primarily affects to Schwann cells, resulting in progressive demyelination (Nelis et al., 1996). Interestingly, oligodendrocytes are much less affected by CMT disease. Indeed, knockout mice for Cx32 show progressive loss of myelin in Schwann cells but not in oligodendrocytes (Scherer et al., 1998). This evidence may imply that Cx32 does not participate in the formation or maintaining of myelin in oligodendrocytes. Alternatively, the latter may reflect that the role of Cx32 in myelin can be greatly bypassed by other connexins expressed in oligodendrocytes (e.g., Cx47). In agreement with these ideas, it has been suggested that Cx45 expression in oligodendrocytes could prevent the demyelination induced by CMT disease (Kunzelmann et al., 1997). In spite of that, the impaired activity observed in neocortical neurons from the Cx32 knockout mouse likely relies on the decreased thickness of the axonal myelin sheaths (Sutor et al., 2000). Therefore, although the involvement of Cx32 in myelin formation is not as evident as in the peripheral nervous system, subclinical manifestations often can be observed (Sutor et al., 2000; Kleopa et al., 2002).

**Table 1:** Brief summary of the involvement of connexins and pannexins in demyelinating diseases.

Disease	Cell type	Connexin or Pannexin	References
Charcot-Marie-Tooth (CMT)	ND	Cx32	Fairweather et al. (1994), Nelis et al. (1996)
	Oligodendrocytes	Cx32	Olympiou et al. (2016)
	Schwann cells and oligodendrocytes	Cx32	Scherer et al. (1998), Kleopa et al. (2002)
	Oligodendrocytes	Cx32 and Cx45	Kunzelmann et al. (1997), Kleopa et al. (2010)
Myelination defects and neuronal hyperexcitability	Schwann cells	Cx32	Sutor et al. (2000)
Pelizaeus-Merzbacher-like (hypomyelinating leukodystrophy 2) and hereditary spastic paraplegia	Oligodendrocytes	Cx47	Osaka et al. (2010), Sargiannidou et al. (2010), Tress et al. (2011), Cotrina and Nedergaard (2012), Zlomuzica et al. (2012)
Oligodendrocyte identity and survival	Oligodendrocytes	Cx47	Schlierf et al. (2006), Pozniak et al. (2010), Takada et al. (2010), Suzuki et al. (2017)
Leukodystrophy	Oligodendrocytes Astrocytes	Cx47	Fasciani et al. (2018)
Multiple sclerosis	Oligodendrocytes	Cx32 and Cx47	Markoullis et al. (2012, 2014)
Experimental autoimmune encephalomyelitis (model of multiple sclerosis)	Oligodendrocytes	Cx32 and Cx47	Constantinescu et al. (2011)
Hypomyelinated leukodystrophy	Oligodendrocytes	Cx32 and Cx47	Wasseff and Scherer (2015)
Demyelination	Oligodendrocytes	Panx1	Hainz et al. (2017)

*This table was intended to show several examples and does not correspond to a compilation of all published evidence. ND, not determined.

The role of oligodendrocyte gap junctional communication in demyelinating diseases is still a matter of ongoing research. Mutations in Cx47 have been found in patients with the central hypomyelinating disorder known as Pelizaeus-Merzbacher-like disease (also known as hypomyelinating leukodystrophy 2) and the hereditary spastic paraplegia (Sargiannidou et al., 2010; Tress et al., 2011; Cotrina and Nedergaard, 2012). In this context, mice bearing a human Cx47^M283T^ missense mutation exhibit a complex age-dependent behavioral phenotype including changes in psychomotor, emotional and memory functions (Zlomuzica et al., 2012). Although with different localizations, both Cx32 and Cx47 are transcriptionally regulated by the same transcription factor: Sox10 (Schlierf et al., 2006), determinant in conferring oligodendrocyte identity and its survival (Pozniak et al., 2010; Takada et al., 2010; Suzuki et al., 2017). Moreover, mutations in the Cx47 gene promoter that binds Sox10 are responsible for the demyelination observed in Pelizaeus-Merzbacher-like disease (Osaka et al., 2010). Of particular interest is the absence of Cx47 in the myelin sheath of oligodendrocytes despite its wide presence in the perikarya (Kleopa et al., 2004; Orthmann-Murphy et al., 2007a), where it form heterotypic GJCs with the Cx43 expressed in astrocytes (Kamasawa et al., 2005). Supporting this notion, when Cx43 is expressed in astrocytes it induces the phosphorylation and further stabilization of Cx47 (May et al., 2013). Similarly, the restrictive permeability of Cx47/Cx43 GJCs is suppressed by a mutation (Cx47^P90S^) linked with leukodystrophy (Fasciani et al., 2018), indicating a new pathogenic mechanism underlying myelin disorders that implies impairments in the oligodendrocyte-astrocyte coupling. Thus, the GJ-mediated crosstalk between these glial cells could be more important than originally thought and deserve further investigation.

Data from animal studies indicates that failures in GJC-mediated coupling may contribute to demyelination (Kleopa et al., 2010; Markoullis et al., 2012, 2014). For instance, a murine model of MS-the experimental autoimmune encephalomyelitis (EAE; Constantinescu et al. (2011))-showed a reduction in GJ plaques composed by both Cx32 or Cx47 in oligodendrocytes within and around lesions but also in the normal appearing white matter (Markoullis et al., 2012). Of note, the latter was paralleled with decreased numbers of Cx43 GJ plaques in astrocytes. A similar loss of GJs formed by Cx32 or Cx47 is observed in oligodendrocytes in the gray matter of post-mortem tissue from MS patients (Markoullis et al., 2014). Instead of what is found in the EAE model, these findings were paralleled with an increase in Cx30 and Cx43 along with an augment of astrocytic GJ plaques, indicating a different molecular profile of astrogliosis in white and gray matter pathology associated to MS. Supporting the role of oligodendrocyte connexins in myelination, ablation of Cx32 and Cx47 generates a phenotype of hypomyelinated leukodystrophy characterized by alterations in gene expression of key enzymes required for the synthesis of myelin lipids, as well as increased expression of genes linked with leukotrienes/prostaglandins synthesis and chemokines/cytokines interactions and signaling pathways (Wasseff and Scherer, 2015). The latter suggests that loss of oligodendrocyte-oligodendrocyte and oligodendrocyte-astrocyte coupling is accompanied by a prominent immune response. Likewise, the knockout mice for Cx32 have a greater sensitivity to inflammatory challenges and cellular stressors (Olympiou et al., 2016), arguing for the relevance of oligodendrocytes GJ signaling in the inflammatory and immune response. Although these findings strongly suggest a role for oligodendrocyte and/or oligodendrocyte-astrocyte coupling in the pathogenesis and progression of demyelinated lesions (see Table 1), functional approaches are necessary to draw definitive conclusions and uncover the underlying mechanisms.

## Hemichannels and Pannexons in Oligodendrocytes

Despite that hemichannels and pannexons were discovered almost three and two decades ago, respectively (Paul et al., 1991; Bruzzone et al., 2003), the available evidence showing their functional expression in oligodendrocytes is still limited and relatively recent. Pioneering work by Niu et al. (2016) showed for first time the presence of functional hemichannels in oligodendrocytes. Using *in vitro* primary cell cultures, they demonstrated that the opening of hemichannels mediates the uptake of a glucose analog in OPCs and oligodendrocytes, this response being more prominent in the former and highly dependent on [Ca^2+^]_i_ (Niu et al., 2016). Importantly, the inhibition of these channels strongly reduced OPC proliferation, suggesting that the uptake of glucose *via* hemichannels is critical for the development of the oligodendroglia lineage. More relevant, when oligodendrocytes are co-cultured with astrocytes knocked-out for Cx43, a decrease in extracellular glucose was found paralleled with a reduction in OPC proliferation (Niu et al., 2016). Therefore, in OPCs, hemichannels may provide a major pathway for glucose entry, which could be supplemented by Cx43-mediated coupling between astrocytes and oligodendrocytes and/or the release of glucose or its metabolites (e.g., lactate) through astrocyte Cx43 hemichannels.

A recent study showed that prenatal exposure to high levels of glucocorticoids increases the dye uptake of oligodendrocytes in brain slices of the offspring (Maturana et al., 2017). This response was eliminated by the application of Gap26, a mimetic peptide that in short periods of exposure (1–15 min) specifically inhibits Cx43 hemichannels rather than Cx43 GJCs. Similar effects were found with A740003, a selective P2X_7_ receptor (P2X_7_R) antagonist, and with the mimetic peptide ^10^panx1, which block Panx1 channels. Given that oligodendrocytes do not express Cx43, the authors explained these results by proposing that high levels of glucocorticoids could increase the activity of Cx43 hemichannels in microglia or astrocytes, consequently increasing the extracellular levels of ATP and positively impacting the opening of Panx1 channels in oligodendrocytes following the stimulation of P2X_7_Rs. In agreement with this idea, the glucocorticoid-induced dye uptake by oligodendrocytes depended on the activation of mast cells and microglia and was accompanied of increased expression of major elements of the inflammasome, including NLRP3, ASC and caspase-1 (Maturana et al., 2017). Despite the above, whether this phenomenon is the result of the upstream activation of Cx43 hemichannels in other brain cells (e.g., microglia, mast cells or astrocytes) remain to be fully elucidated.

The functional expression of Panx1 channels in oligodendrocytes was first described by Domercq et al. (2010). Using primary cultures of oligodendrocytes, they demonstrated that oxygen and glucose deprivation (OGD) increases ATP release and ischemic ionic imbalance-induced cell death by a mechanism involving the activation of P2X_7_Rs and Panx1 channels (Figure 2; Domercq et al., 2010). These findings were paralleled with increased intracellular Ca^2+^ concentration ([Ca^2+^]_i_), which agrees with the fact that high extracellular ATP may trigger a rise of cytosolic Ca^2+^ in oligodendrocytes by activating P2XRs (James and Butt, 2001). P2X_7_R stimulation may lead to ATP release in oligodendrocytes through at least one mechanism implicating hemichannels and/or pannexons. Despite that P2X_7_R activation rises [Ca^2+^]_i_ (Baroja-Mazo et al., 2013) and opens Cx43 hemichannels and Panx1 channels (Locovei et al., 2006; De Bock et al., 2012), the Panx1-mediated release of ATP depends on protein-protein interactions between P2X_7_Rs and Panx1 (Locovei et al., 2007). Consistent with this notion, Panx1 co-immunoprecipitates with P2X_7_Rs (Silverman et al., 2009; Poornima et al., 2012), where the proline 451 in the C-terminal tails of these receptors has been involved (Iglesias et al., 2008; Sorge et al., 2012). The positive feedback loop of activation among P2X_7_Rs and hemichannels/pannexons could explain the oligodendrocyte damage occurring in EAE, where high release of ATP has been linked to P2XR activation (Matute et al., 2007). Supporting this line of thought, probenecid, a Panx1 channel blocker, drastically prevent inflammation and oligodendrocyte damage triggered by cuprizone diet, a well-known model of demyelination (Hainz et al., 2017).

Further studies are necessary for elucidating the nature of the connexins involved in the activity of oligodendrocyte hemichannels, as well as how these channels and pannexons participate in the pathogenesis of diseases characterized by demyelination and oligodendrocyte dysfunction.

## Conclusions

Oligodendrocytes serve to maintain critical brain functions such as myelination, metabolic supply, ion buffering, and recent findings suggest also a possible contribution to setting neuronal bursting properties. The evidence discussed in this review indicates that both oligodendrocyte-oligodendrocyte coupling (mainly mediated by Cx47 and Cx32 homotypic GJCs) and oligodendrocyte-astrocyte coupling (mediated by Cx47/Cx43 or Cx32/Cx30 pairs) play a pivotal role in forming a functional panglial syncytium supporting the buffering of extracellular K^+^ and metabolic supply to axons. Although the available literature suggests that oligodendrocyte progenitors would not contribute to this functional syncytial network, their role could not be completely ruled out based on recent findings showing the coupling of OPCs with astrocytes through Cx47 (Liu et al., 2017; Xu et al., 2017). Future research will elucidate whether OPCs make part of this interconnected glial network. Of interest is the fact that both OPCs and mature oligodendrocytes are endowed with hemichannels that allow the passage of relevant bioactive molecules (e.g., glucose), which might be involved not only in oligodendroglia maturation, but also in metabolic coupling and energy supply to neurons.

The fact that patients with demyelinating diseases (i.e., MS) exhibits anomalies in normal expression and distribution of brain connexins, pointing out a specific role for hemichannels and GJCs in these neuropathologies. The studies reviewed here strength the recent body of evidence supporting a dysregulation of hemichannels and GJCs in glial cell types as a common phenomenon to several neurogenerative diseases. Future investigation should uncover the contribution of connexin- and pannexin-based channels to the onset and progression of demyelinating diseases.

## Author Contributions

JAO, MR and FCO conceived and designed the major ideas developed in the manuscript. JAO designed the Figure 2. All authors wrote and edited the manuscript. All authors read and approved the final manuscript.

## Conflict of Interest Statement

The authors declare that the research was conducted in the absence of any commercial or financial relationships that could be construed as a potential conflict of interest.
